# Autophagy Determines Distinct Cell Fates in Human Amnion and Chorion Cells

**DOI:** 10.1080/27694127.2024.2306086

**Published:** 2024-02-07

**Authors:** Mary Elise L. Severino, Lauren S. Richardson, Ananth Kumar Kammala, Enkhtuya Radnaa, Kamil Khanipov, Leslie Michelle M. Dalmacio, Indira U. Mysorekar, Marian Kacerovsky, Ramkumar Menon

**Affiliations:** a Division of Basic Science & Translational Research, Department of Obstetrics and Gynecology, The University of Texas Medical Branch at Galveston, Texas, USA; b College of Medicine, University of the Philippines Manila, Manila, Philippines; c Department of Pharmacology and Toxicology, The University of Texas Medical Branch at Galveston, Texas, USA; d Department of Medicine, Section of Infectious Diseases, Baylor College of Medicine, Houston, TX, USA; e Department of Molecular Virology and Microbiology, Baylor College of Medicine, Houston, TX, USA; f Huffington Centre on Aging, Baylor College of Medicine, Houston, TX, USA; g Department of Obstetrics and Gynecology, University Hospital Hradec Kralove, Faculty of Medicine in Hradec Kralove, Charles University, Hradec Kralove, Czechia

**Keywords:** Membrane rupture, Oxidative stress, p38 MAPK, chorion, trophoblast, senescence

## Abstract

Human fetal membranes (amniochorion) that line the intrauterine cavity consist of two distinct cell layers; single-layer amnion epithelial cells (AEC) and multilayer chorion trophoblast cells (CTC). These layers are connected through a collagen-rich extracellular matrix. Cellular remodeling helps support membrane growth and integrity during gestation and helps to maintain pregnancy. Preterm prelabor rupture of the human amniochorionic (fetal) membrane (pPROM) is antecedent to 40% of all spontaneous preterm birth. Oxidative stress (OS) induced activation of the p38 MAPK due to various maternal risk exposures and the amniochorion cells’ senescence are reported pathological features of pPROM. Our transcriptomics analysis implicated dysregulated autophagy and epithelial-mesenchymal transition (EMT) in fetal membranes from pPROM. The molecular interplay between OS-induced p38 MAPK activation, autophagy, and EMT was investigated in AECs and CTCs to better understand the involvement of autophagy and EMT. We report the differential impact of OS on the autophagic machinery in AECs and CTCs, resulting in distinct cell fates. In AECs, OS-induced p38 MAPK activation causes autophagosome accumulation and reduced autophagic flux mediated by decreased ULK1 activity and kinase activity, leading to senescence. In CTCs, induction of autophagy has a limited effect; however, inhibition of autophagy led to SQSTM1-mediated EMT of trophoblast cells. Autophagy, EMT, and senescence were associated with proinflammatory changes. Thus, AECs and CTCs respond differently to OS via differential autophagy response, partly mediated via p38 MAPK. Besides senescence, OS-induced autophagy dysregulation in amniochorion cells may play a mechanistic role in pPROM pathophysiology.

## Introduction

The fetal membranes is vital in maintaining homeostatic balance in the presence of constant challenges (immune, structural, mechanical, and endocrine) during pregnancy [1]. They are composed of amnion epithelial cells (AEC), a single-cell epithelial layer close to the fetus, and the chorion trophoblast cell (CTC) layer that forms the feto-maternal interface barrier connected to the maternal decidua. The two layers are connected by a collagen-rich extracellular matrix that provides a structural framework of the membranes and contains mesenchymal cells [2–5]. During pregnancy, fetal membranes undergo a telomere-dependent, p38 mitogen-activated protein kinase (MAPK)-mediated senescence, and produce senescence-associated secretory phenotype (SASP) [6]. Senescence is accelerated at term by oxidative stress (OS) [7–11]. Senescent and dysfunctional fetal membranes lead to an inflammatory overload that shifts the quiescence status of the intrauterine cavity into an active form initiating parturition [1]. Untimely activation of the p38 MAPK-mediated signaling or inappropriate inflammatory imbalance can lead to preterm labor and preterm premature rupture of membranes (pPROM) leading to adverse outcomes for both mothers and neonates [12–14].

Autophagy is an evolutionarily conserved catalytic process that plays a crucial role in maintaining redox balance in cells. It is a lysosome-dependent intracellular degradation system by which cytoplasmic components including damaged macromolecules and organelles are degraded, thereby providing the new building blocks for cellular recycling [15–17]. This process is tightly regulated by several autophagy-related genes that can be classified into four groups, namely autophagy core genes, autophagy regulators, lysosome-related genes and mTOR pathway-related genes [18]. It also modulates antioxidant defense against OS and facilitate elimination of damaged proteins and organelles [19–21]. Increased ROS production and/or decreased antioxidant defenses results in oxidative damage of lipids, proteins, and nucleic acids [22,23]. ROS activate the autophagy pathway as a cytoprotective negative-feedback mechanism that selectively eliminates sources of ROS production, such as dysfunctional mitochondria and peroxisomes thus preventing oxidative damage [24]. Dysregulation of autophagy has been implicated in the pathogenesis of a wide range of human diseases [25].

Accumulation of damaged organelles in the fetal membranes under OS raises the question of whether autophagy is activated or inhibited during membrane rupture. In this regard, limited studies on autophagy in fetal membranes suggest that there is decreased autophagy in preterm and term laboring membranes [26]. With OS as key mediator of senescence in fetal membranes, we postulate that autophagy may play a key role in the regulation of oxidative-stress induced senescence in fetal membranes. Since autophagy and senescence are critical cellular responses to stress, such as DNA damage and OS, exploring their relationship may help us better understand fetal membrane homeostasis during pregnancy.

Here, we provide evidence that distinct autophagy-related gene and pathways are modulated in term laboring membranes and pPROM. Mechanistically, we show that autophagy induction in AECs leads to senescence while autophagy inhibition in CTCs lead to EMT, which are mediated by p38 MAPK, underscoring a potential role of autophagy in maintaining fetal membrane homeostasis. Taken together, our data strongly suggests that autophagy is dysregulated in fetal membrane rupture and has a mechanistic role in the pathophysiology of pPROM.

## Results

### Distinct autophagy profiles of fetal membranes from term labor/not in labor and pPROM

**Figure 1.**
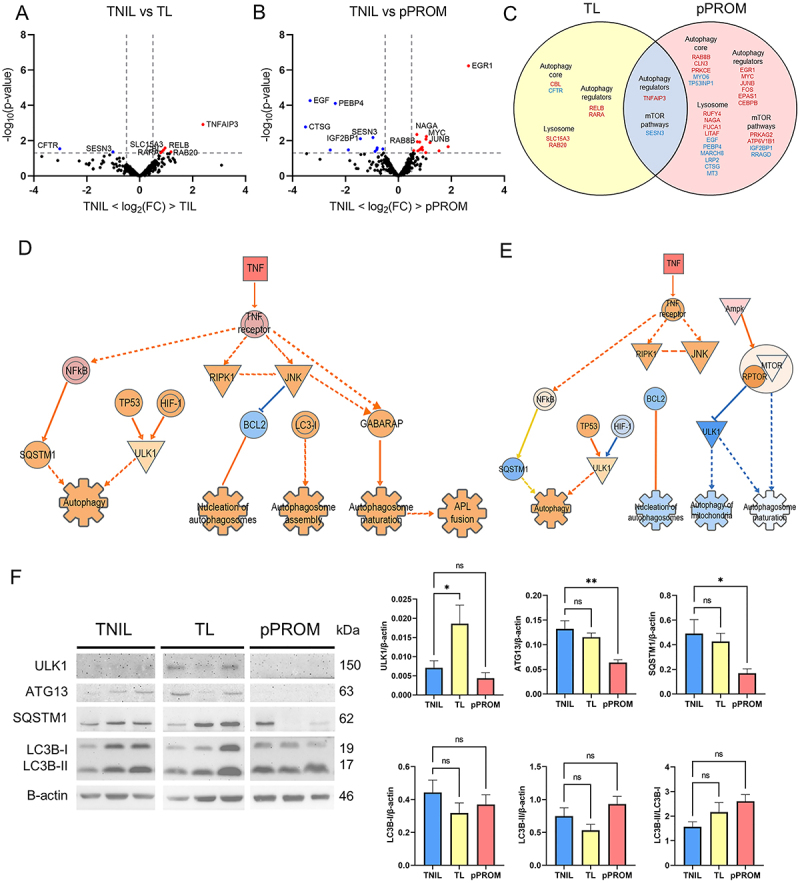
Fetal membranes from different pregnancy conditions have distinct autophagy profiles. Volcano plots depicting differentially expressed autophagy genes in fetal membranes of (A) term in labor (TL, n = 6), or (B) preterm premature rupture of membranes (pPROM, n = 12) compared to term not in labor (TNIL, n = 3) showing autophagy genes that are significantly upregulated (red) or downregulated (blue) between comparison groups. Dual thresholding of *p*-value < 0.05 and |log2 fold change| > 0.5 was used to determine significant differential expression (shown as gray dashed lines). (C) Venn diagram showing the similarities and differences in the differentially expressed autophagy genes. Genes that are upregulated are shown in red font while those downregulated are shown in blue font. (D) Ingenuity pathway analysis predicts activation of autophagy pathways in fetal membranes of TL compared to TNIL. (E) Ingenuity pathway analysis predicts inhibition of some autophagy functions in fetal membranes of pPROM compared to TNIL. Legend: orange = predicted activation, blue = predicted inhibition, red = increased gene expression. (F) Representative immunoblots are shown and quantification analysis of autophagy proteins in fetal membranes of TNIL (n = 13), TL (n = 13) and pPROM (n = 5) was done. β-actin was used as an internal standard for protein loading. The values are presented as means ± SEM (*: *p* < 0.05, **: p < 0.01, ns: not significant).

To determine whether expression of autophagy genes in fetal membranes was altered in labor, we examined fetal membrane tissue samples from term not in labor (TNIL, n = 3), term in labor (TL, n = 6), and pPROM (n = 12) using RNA sequencing. We analyzed pairwise comparison of TL and pPROM membranes to non-laboring membranes from TNIL to investigate the impact of distinct labor-associated processes. Differential expression analyses of TNIL vs TL revealed 8 autophagy related DEGs (Figure 1A). Positive regulators of autophagy genes *TNFAIP3, RELB*, and *RARA* were upregulated in TL membranes. Selective autophagy cargo receptor *CBL* and lysosomal genes *SLC15A3* and *RAB20* showed increased expression. Conversely, the mTOR regulator *SESN3* and autophagy core gene *CFTR* were downregulated in TL membranes. TNIL vs pPROM comparison revealed 27 autophagy-related *DEGs* (Figure 1B). Autophagy regulators *EGR1, MYC, JUNB, FOS, EPAS1* and *CEBPB* were upregulated in the pPROM group compared to non-laboring membranes (Figure 1C). All these are positive regulators of autophagy except for *JUNB*, which is a negative regulator. Six out of ten lysosomal genes were downregulated while three out of five of the mTOR pathway-related genes were also downregulated. Only *TNFAIP3* and *SESN3* are the common genes between TL and pPROM, which are positive regulator of autophagy and mTOR regulator, respectively (Figure 1C). Overall, results show that selected autophagy genes are modulated between TL and pPROM.

Next, to identify autophagy signaling pathways involved in TL and pPROM, prediction of canonical pathways and interaction network molecules were executed using Ingenuity Pathway Analysis (IPA®, Qiagen). We examined the Autophagy Canonical Pathway to determine which genes within the pathway changed in expression and altered downstream signaling. Fetal membranes from TL predicted activation of the autophagy pathway through proinflammatory TNF activation which causes downstream upregulation of cell survival pathways of RIPK1 including NF-κB and JNK pathways (Figure 1D; Supplementary Figure 1). In fetal membranes we show modulators of genotoxic stress TP53 and of hypoxia HIF-1, that have been shown to be major activators of autophagy. Together, our analysis suggests normal term labor activates the autophagy pathway (*p* = 0.00026, z score = 1.633). In contrast, we note that pPROM samples, there are multiple autophagy processes ‘autophagosome maturation’ and ’mitophagy’ are reduced (Figure 1E; Supplemental Figure 1).

### Effect of term in labor and preterm premature rupture of membranes on the expression of autophagy proteins in fetal membranes

Next, to validate our findings from the ingenuity analysis and confirm impact on expression due to labor or pPROM we selected four canonical autophagy-pathway proteins to. 1) ULK1 – a protein essential for autophagy induction [27,28], 2) ATG13 – a protein adapter in the ULK1 complex that enhances ULK1 kinase activity [29], 3) p62/SQSTM1– a selective autophagy cargo receptor [30], and 4) LC3B – a central protein in the autophagy pathway [28,31]. Protein expression levels in fetal membranes were assessed by western blot analysis in TNIL (n = 13), TL (n = 13), and pPROM (n = 5). We note increased expression of ULK1 in TL compared to TNIL (*p* = 0.038) but no significant changes in ATG13, and SQSTM1 were noted. In contrast, there was a decrease in SQSTM1 (*p* = 0.042) and ATG13 (*p* = 0.007) levels in pPROM compared to TNIL but no difference in ULK1 (Figure 1F). There was no significant difference in the free non-lipidated form LC3B-I, membrane bound LC3B-II as well as the ratio between the two (LC3B-II/LC3B-I) in TL and pPROM compared to TNIL. These findings suggest that autophagy process may be activated in laboring membranes.

### OS decreases autophagic flux in AECs while increases in CTCs

The role of OS in fetal membrane rupture has been well-established, but its effects on autophagy in this process have not yet been investigated. To understand the mechanism of how OS affects autophagic response in fetal membranes, we used two cell lines, namely AECs and CTCs previously isolated from term not in labor fetal membrane layers and immortalized [32]. We induced OS at using cigarette smoke extract 6-, 12- and 24-hour time points within AEC and CTC cultures. We used water soluble fractions of cigarette smoke extract (CSE) as an inducer of OS as previously reported [33].

**Figure 2.**
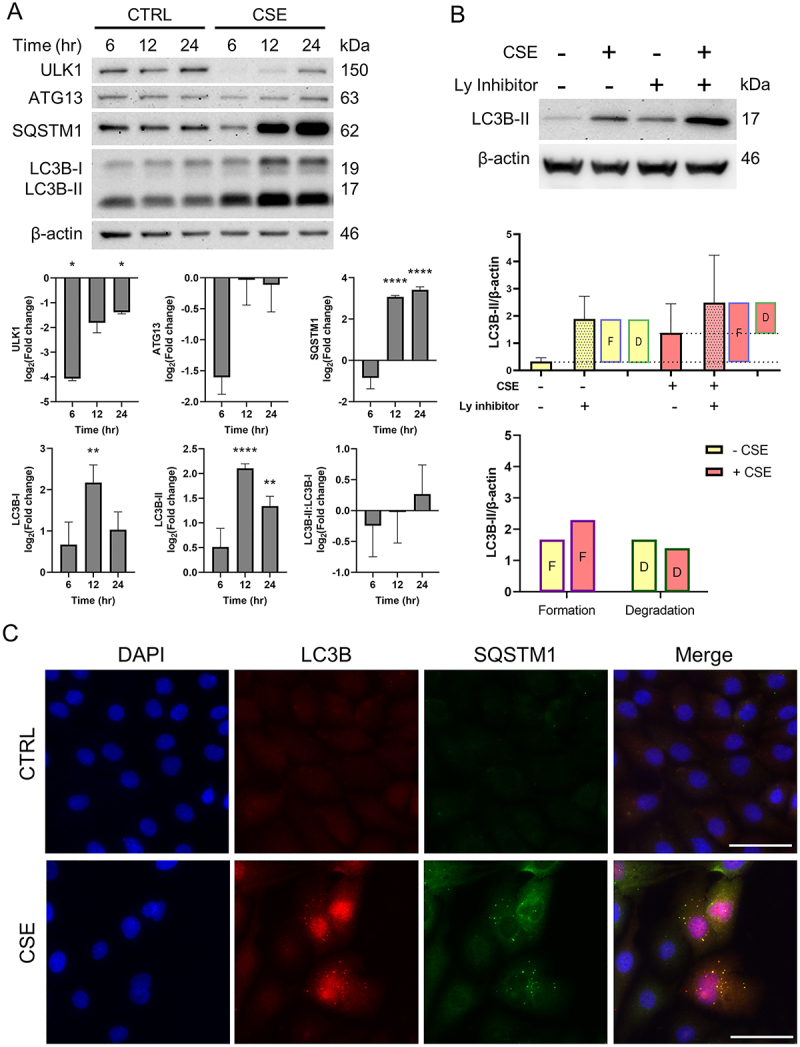
Oxidative stress decreases autophagic flux by cargo and autophagosome buildup in amnion epithelial cells.

We found that in AECs subjected to OS, ULK1 was downregulated compared to control (p values 6hr: 0.021, 12hr: 0.071, 24hr: 0.030) (Figure 2A). ATG13 remained unchanged in all treatment periods. OS did not change SQSTM1 expression after short exposure to CSE (6 hr); however, it accumulated at longer exposure periods which may reflect cargo accumulation with prolonged exposure to OS (*p* values 12hr: < 0.001, 24hr: < 0.001). A similar trend could be seen with LC3B-I (p value 12hr: 0.005) and LC3B-II (*p* values 12hr: < 0.001, 24hr: < 0.001). The accumulation of autophagosomes in AEC is supported by an increase in autophagosome marker LC3B and cargo receptor SQSTM1. These findings indicate the autophagosome accumulation occurs in AECs (Figure 2A).

(A) AECs were exposed to cigarette smoke extract (CSE) for 6hr, 12hr, and 24hr periods. Western blot analysis and quantification revealed an initial decline in ULK1 levels which increased over time. No changes in ATG13 levels were observed. SQSTM1, LC3B-I and LC3B-II levels showed no initial change after 6 hr exposure but accumulated at 12 hr exposure. LC3B-II:LC3B-I ratio remained unchanged. Bar graphs presented as log_2_(Fold change) as ratio of treatment over control. Error bars represent mean ± SEM. Statistical analysis done on pairwise comparison of treatment and corresponding control. (*: *p* < 0.05, **: *p* < 0.01, ****: *p* < 0.0001) (B) Western blot analysis was done to measure changes in LC3B-II levels after 24 hour exposure to CSE and/or lysosomal inhibitor bafilomycin A1 for 24 hours. Analysis showed the amount of autophagosomes formed (F) and degraded (D) in both normal and OS conditions. Differences in levels were compared and showed that there is net increase in autophagosomes which implies accumulation. Results imply that OS decreased autophagic flux in AECs. (C) Immunocytochemistry staining of LC3B and SQSTM1 were done to visualize autophagosome formation in AECs under OS (CSE) after 48 hour exposure. OS induced a punctate staining of LC3B and SQSTM1 representing autophagosome formation. Scale bar = 50 μm (Blue: DAPI, red: LC3B, green: SQSTM1).

Changes in the LC3B levels may either be from changes in autophagy induction or degradation of autophagosome by lysosomes. To monitor autophagic flux, we measured the degradation of LC3B in lysosomes by comparing the amount of LC3B-II in cells treated with and without lysosomal inhibitor (bafilomycin A1). Western blot analysis and graphs representing the amount of LC3B-II degradation and formation are shown in Figure 2B. The difference between control groups with and without lysosomal inhibitors corresponds to the amount of autophagosomes formed at baseline levels (Figure 2B, top bar graph). During normal conditions, there is autophagic equilibrium where the amount of autophagosomes formed is equal to those degraded. The difference in levels between OS (CSE treatment) with lysosomal inhibitor and control without lysosomal inhibitors corresponds to the total amount of autophagosomes formed. The differences between OS with and without lysosomal inhibitor corresponds to the amount of autophagosomes degraded during OS conditions.

Autophagic flux analysis by western blot revealed no significant changes in levels of autophagosome formation and degradation (Figure 2B). Accumulation of autophagosomes were visualized under fluorescence microscopy OS in cells showed SQSTM1 and LC3B puncta that correspond to autophagosome formation (Figure 2C). Together, our results suggest that OS decreases autophagic flux in AECs, thus leading to autophagosome accumulation.

### OS increases autophagic flux in CTC

**Figure 3.**
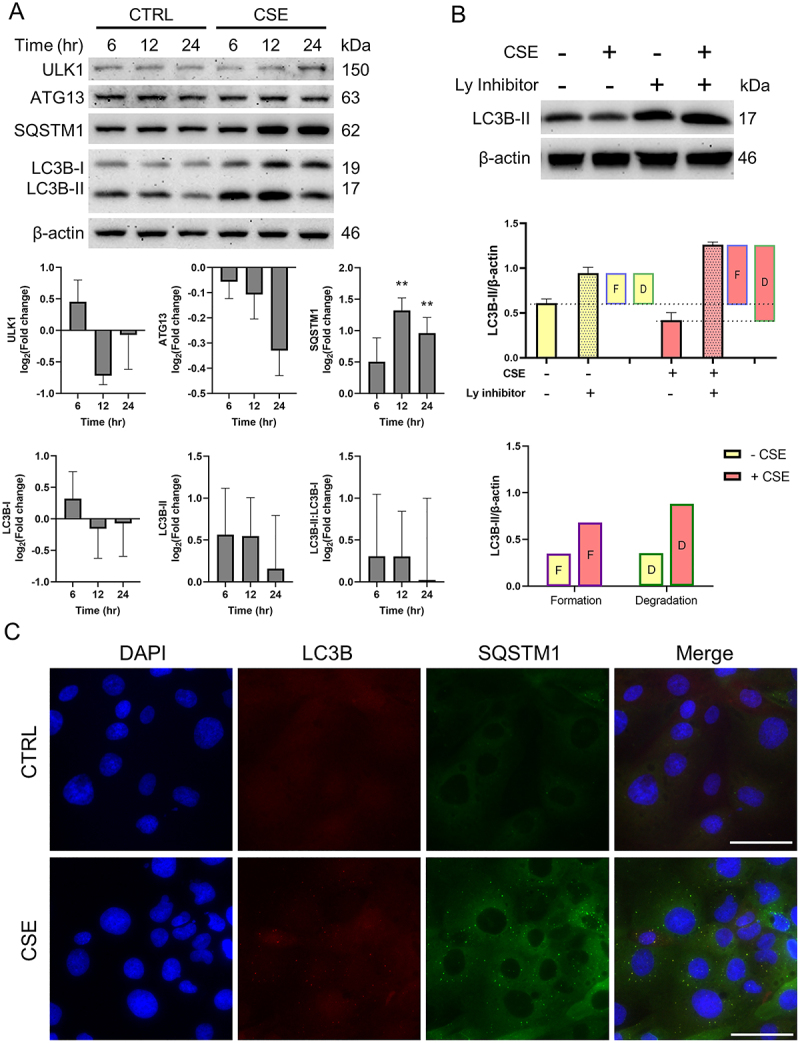
Oxidative stress increases autophagic flux by increasing autophagosome consumption in chorion trophoblast cells.

We next examined the impact on CTCs. In contrast to AECs, we found that OS in CTCs showed no changes in ULK1, ATG13 and LC3B levels while SQSTM1 levels were increased (*p* values 12hr: 0.002, 24hr: 0.007) (Figure 3A). These findings support our hypothesis that CTCs, as a major barrier at the feto-maternal interface, are able to maintain homeostasis in response to OS.

(A) CTCs were exposed to cigarette smoke extract (CSE) for 6hr, 12hr, and 24hr periods. Western blot analysis and quantification revealed no changes in ULK1 and ATG13 levels. SQSTM1 levels were elevated starting at 12 hour exposure. LC3B-I and LC3B-II levels were unchanged over time. LC3B-II:LC3B-I ratio also remained unchanged. Bar graphs presented as log_2_(Fold change) as ratio of treatment over control. Error bars represent mean ± SEM. Statistical analysis done on pairwise comparison of treatment and corresponding control. (**: *p* < 0.01) (B) Western blot analysis was done to measure changes in LC3B-II levels after 24 hour exposure to CSE and/or lysosomal inhibitor bafilomycin A1 for 24 hours. Analysis showed the amount of autophagosomes formed (F) and degraded (D) in both normal and OS conditions. Differences in levels were compared and showed that there is net decrease in autophagosomes which implies increased consumption. Results imply that OS increased autophagic flux in CTCs. (C) Immunocytochemistry staining of LC3B and SQSTM1 were done to visualize autophagosome formation in CTCs under OS (CSE) after 48 hour exposure. OS induced a punctate staining of SQSTM1 but no LC3B staining. Scale bar = 50 μm (Blue: DAPI, red: LC3B, green: SQSTM1).

Comparisons of LC3B-II levels in OS-induced CTCs with or without a lysosomal inhibitor (Bafilomycin A1) revealed no increase in autophagosome formation but a significant increase in degradation (*p* = 0.006) (Figure 3B). Fluorescence microscopic analysis of OS-treated CTCs showed an absence of LC3B puncta staining but an increase in SQSTM1 (Figure 3C). Overall, these findings suggest an increase in autophagic flux in CTCs in response to OS as opposed to a decrease in autophagy flux in AECs.

### p38 MAPK mediates the changes in autophagy regulation in AECs

**Figure 4.**
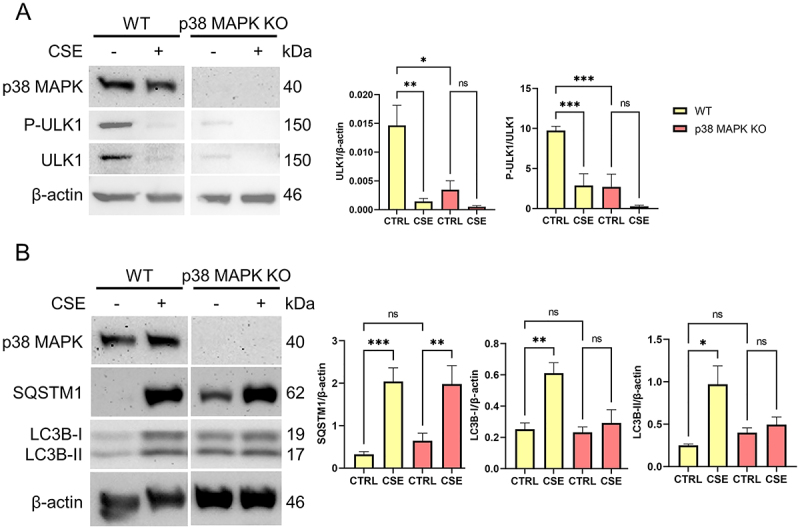
p38 MAPK mediates ULK1 activation and autophagosome formation in amnion epithelial cells.

p38 MAPK is implicated in both OS induced senescence and EMT of the fetal membrane cells [10,11,34]. Next, we investigated whether p38 MAPK has a role in the differential autophagic response in AECs and CTS. We used a p38 MAPK knockout in AEC cell lines generated by previous studies [10,11]. In normal conditions, ULK1 is phosphorylated in activation site Ser555 in AECs (Figure 4A). When AECs were exposed to OS, both P-ULK1 (*p* < 0.001) and ULK1 (*p* = 0.005) levels were decreased. In p38 MAPK KO cells, baseline levels of P-ULK1 (*p* < 0.001) and ULK1 (*p* = 0.013) were decreased. SQSTM1 levels were unchanged in both wild type and p38 MAPK knockout cells (Figure 4B). However, OS-induced increase in LC3B-I and LC3B-II were no longer observed in p38 MAPK knockout cells. Our results imply upon OS treatment in AECs, p38 MAPK dependent autophagy is initiated by ULK1 activation.

(A) Western blot analysis showed sustained levels of ULK1 activation by Ser555 phosphorylation after 3 hour exposure to cigarette smoke extract (CSE). OS by CSE exposure decreases activation of ULK1. Silencing p38 MAPK decreased baseline P-ULK1. Findings imply that p38 MAPK mediates ULK1 activation in AECs. (B) Western blot analysis showed no changes in SQSTM1 levels with wild type and p38 MAPK knockout (KO) AECs after 24 hour exposure to CSE. OS-induced increase in LC3B-I and LC3B-II were no longer observed in p38 MAPK KO AECs. Findings suggest that p38 MAPK mediates ULK1 activation in normal conditions and is involved in autophagosomes formation in AECs. Error bars represent mean ± SEM. (*: *p* < 0.05, **: *p* < 0.01, ***: *p* < 0.001, ns: not significant).

### p38 MAPK mediates SQSTM1 increase in CTCs

**Figure 5.**
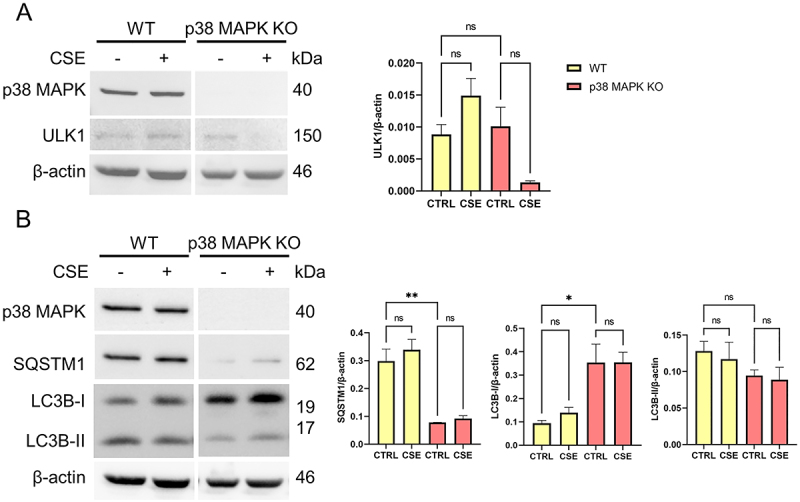
p38 MAPK mediates SQSTM1 expression and LC3B recruitment into autophagosomes in chorion trophoblast cells.

To determine whether p38 MAPK also mediates the autophagic response in CTCs, we compared levels of autophagy markers in p38 MAPK knockout cells compared to wild type CTCs. There were no changes observed in ULK1 levels in wild type and p38 MAPK knockout cells (Figure 5A). Phosphorylation of ULK1 at Ser555 was not observed in CTCs (Supplementary Figure 2). However, SQSTM1 levels were decreased (*p* = 0.002) while LC3B-I levels were elevated (*p* = 0.031) in p38 MAPK knockout cells compared to wild type (Figure 5B). Data suggests that p38 MAPK mediates SQSTM1 expression and its likely involvement in LC3B recruitment into autophagosomes in CTCs.

(A) Western blot analysis showed no changes in ULK1 levels in wild type (WT) and p38 MAPK KO cells after 3 hour exposure to cigarette smoke extract (CSE). (B) Western blot analysis showed decrease in SQSTM1 and increase in LC3B-I levels in p38 MAPK KO cells after 24 hour exposure to CSE. No changes were observed in LC3B-II levels. Findings suggest that p38 MAPK mediates SQSTM1 expression and recruitment of LC3B into autophagosomes in CTCs. Error bars represent mean ± SEM. (*: *p* < 0.05, **: *p* < 0.01, ns: not significant).

### P-p38 MAPK is associated with decrease in ULK1 activation and kinase activity in AECs

**Figure 6.**
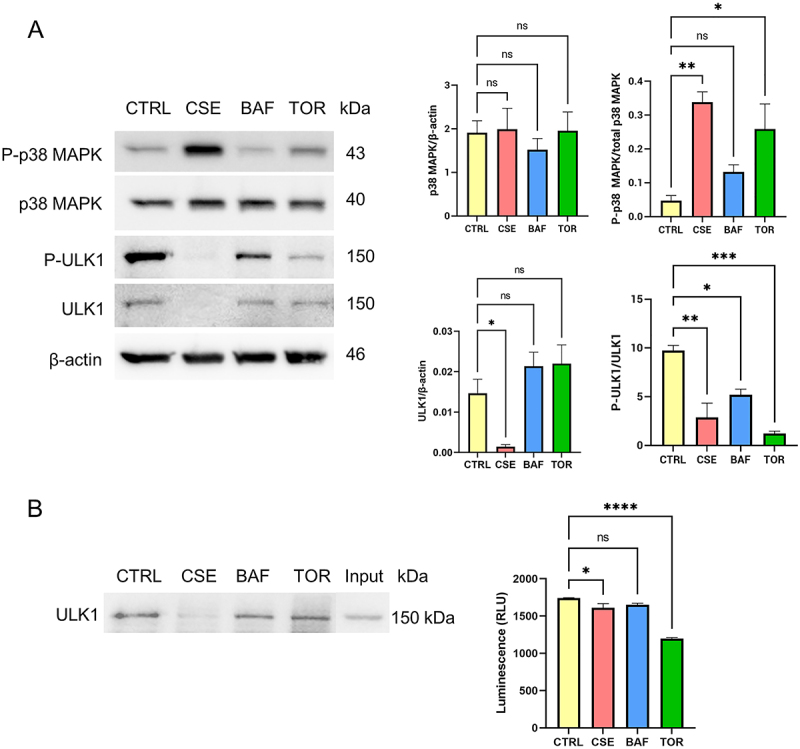
Oxidative stress and induction of autophagy lead to the activation of p38 MAPK and a concomitant decrease in ULK1 activation in amnion epithelial cells.

OS is known to increase phosphorylation of p38 MAPK [35]. Here we compared the changes in ULK1 elicited by OS to autophagy inhibition by bafilomycin A1 treatment (100 nM), and to autophagy induction by torin-1 treatment (30 nM). Bafilomycin A1 inhibits autophagy by increasing lysosomal pH thereby blocking the fusion of autophagosomes with lysosomes, resulting to accumulation of autophagosomes. Torin-1 induces autophagy by inhibiting mTORC1 and mTORC2 which are inhibitors of ULK1 phosphorylation. Our data showed that both OS (*p* = 0.003) and autophagy induction (*p* = 0.019) increases P-p38 MAPK in AECs (Figure 6A). Interestingly, P-ULK1 was decreased in all treatments (p values CSE: 0.001, BAF: 0.016, TOR: < 0.001), while total levels of ULK1 were decreased only in CSE-treated cells (*p* = 0.025). To assess the effects on ULK1 kinase activity, we immunoprecipitated ULK1 and used non-radioactive approach to determine the kinase activity. OS (*p* = 0.042) and autophagy induction (*p* < 0.001) decreased in ULK1 kinase activity, which corresponds to the decrease in P-ULK1 levels (Figure 6B). Thus, P-p38 MAPK is associated with decrease in ULK1 activation and kinase activity in AECs. In contrast, OS and autophagy induction did not have any effect on ULK1 levels and activity in CTCs (Supplementary Figure 3).

(A) Western blot analysis and quantification was done to assess activation of p38 MAPK and ULK1 under 3 hour exposure to OS by cigarette smoke extract (CSE), autophagy inhibition by bafilomycin A1 (BAF) and autophagy induction by torin-1 (TOR). OS and autophagy induction caused increase in p38 MAPK activation by P-p38 MAPK. Total p38 MAPK levels were unchanged. OS and autophagy induction decreased P-ULK1 activation at Ser555. (B) ULK1 was immunoprecipitated and kinase activity was assessed. ULK1 kinase activity were decreased in OS and autophagy induction. Error bars represent mean ± SEM. (*: *p* < 0.05, **: *p* < 0.01, ****: *p* < 0.0001, ns: not significant, CSE: cigarette smoke extract, BAF: Bafilomycin A1, TOR: Torin-1)

### p38 MAPK mediates senescence by OS and autophagy induction in AECs

**Figure 7.**
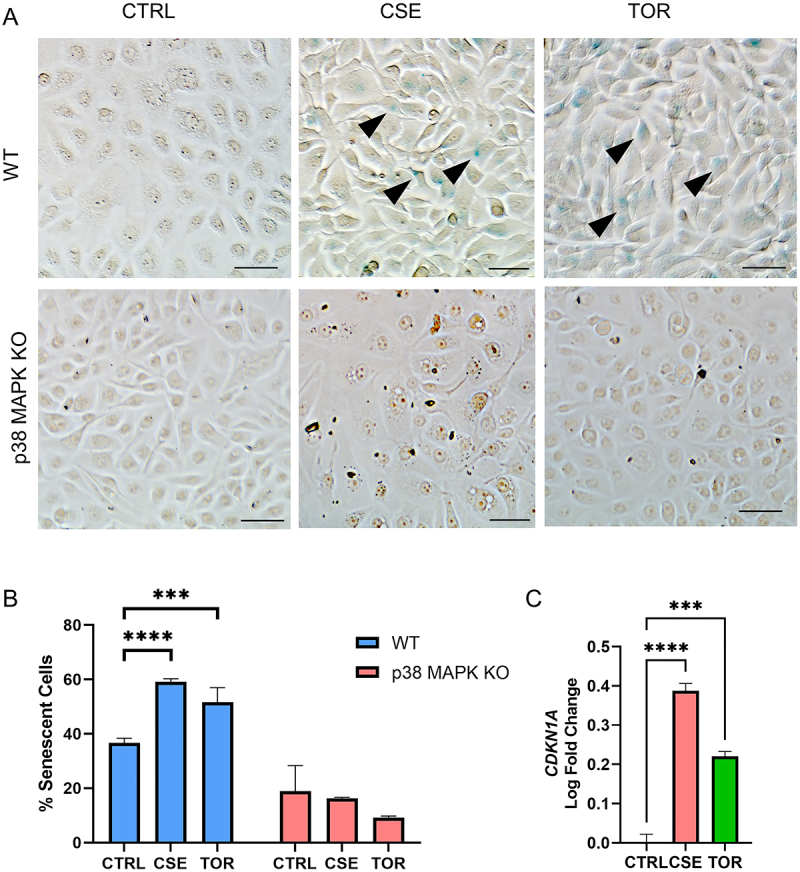
p38 MAPK mediates OS and autophagy induction-induced senescence in amnion epithelial cells.

p38 MAPK plays a central role in cell cycle arrest and senescence in the fetal membranes [10,11]. OS is known to increase senescence in AECs; however, the effect of autophagy inhibition or induction on senescence is not yet known in this compartment. Histochemical staining for beta-galactosidase, a marker of senescence, staining was seen in cells after autophagy induction via Torin1 treatment (Figure 7A). Quantitative measurement of senescence by flow cytometry showed increase in senescence-associated beta-galactosidase activity in AECs under OS (*p* < 0.001) and autophagy induction (*p* < 0.001) (Figure 7B). These are associated by increase in transcription of CDKN1A (*p* values CSE: < 0.001, TOR: < 0.001), which encodes for p21, a senescence mediator (Figure 7C). Interestingly, senescent cells were no longer observed in p38 MAPK knockout cell lines. This suggests that p38 MAPK mediates senescence by OS and autophagy induction in AECs. In contrast, autophagy induction does not induce senescence in CTCs (Supplementary Figure 4).

Senescent were detected in AECs under OS (CSE) and autophagy induction (TOR) after 48 hours of exposure. (A) Histochemical detection of senescence-associated beta-galactosidase (SA-β-Gal) activity exhibited blue staining in senescent cells (arrowheads) upon OS and autophagy induction. Knockout of p38 MAPK showed no development of senescence in both CSE- and torin-1 treated cells. Scale bar = 50 μm. (B) Senescence flow cytometry quantitatively measured SA-β-Gal activity. Results showed increase in senescent cells in AECs under OS and autophagy inhibition. p38 MAPK knockout cell lines do not exhibit increase in senescent cells upon OS and autophagy induction. (C) qRT-PCR analysis showed increase in CDKN1A transcription upon OS and autophagy induction. Error bars represent mean ± SEM. (***: *p* < 0.001, ****: *p* < 0.0001, BAF: Bafilomycin A1, TOR: Torin-1, CSE: cigarette smoke extract).

### EMT induced by autophagy inhibition in CTC is mediated by SQSTM1

**Figure 8.**
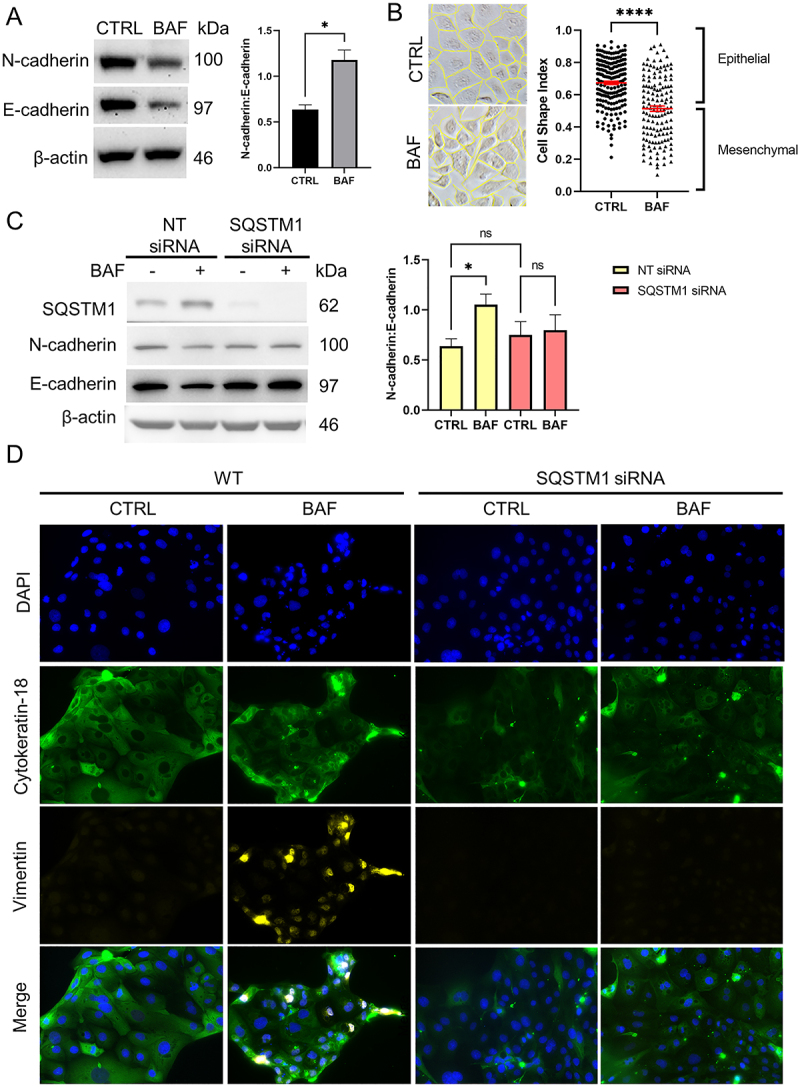
SQSTM1 mediates autophagy inhibition-induced epithelial-mesenchymal transition in chorion trophoblast cells.

While OS is known to induce irreversible EMT in AECs [36], EMT in CTCs has not yet been well-studied. We demonstrated for the first time the induction of EMT in CTCs through the inhibition of autophagy (Figure 8). We blocked autophagy using bafilomycin A1 treatment in CTCs and found that it increased N-cadherin:E-cadherin ratio (*p* = 0.011) (Figure 8A,C), shifted to a mesenchymal morphology by decreased cell shape index (*p* < 0.001) (Figure 8B), and increased expression of mesenchymal marker vimentin (Figure 8D). To determine whether SQSTM1-selective autophagy mediates the EMT induced by autophagy inhibition, we silenced SQSTM1 using short interfering RNA (siRNA) and assessed N-cadherin:E-cadherin ratio, and vimentin staining by immunocytochemistry. Knockdown was validated using western blot. Upon western blot analysis, the increase in N-cadherin:E-cadherin ratio by autophagy inhibition was no longer observed in SQSTM1 knockdown (Figure 8C). Additionally, mesenchymal marker vimentin staining, which was observed in autophagy inhibition of wild type CTCs was not seen in SQSTM1 knockdown cells (Figure 8D). This suggests that EMT by autophagy inhibition in CTS is mediated by SQSTM1. In contrast, autophagy inhibition did not induce EMT in AECs (Supplementary Figure 5).

(A) Western blot analysis showed increase in N-cadherin:E-cadherin ratio upon autophagy inhibition in CTCs for 48 hours. (B) Cell shape index analysis show shift to mesenchymal morphology upon autophagy inhibition. siRNA was used to knockdown SQSTM1 in CTCs. Non-targeting (NT) siRNA was used as control. (C) Western blot analysis showed that silencing of SQSTM1 negated the autophagy inhibition-induced increase in N-cadherin:E-cadherin ratio. Error bars represent mean ± SEM. (*: *p* < 0.05, ns: not significant, BAF: Bafilomycin A1). (D) Immunocytochemistry showed vimentin expression in autophagy inhibition in wild type (WT) cells after 48 hours. Silencing SQSTM1 eliminated autophagy inhibition-induced vimentin expression. This implies autophagy inhibition-induced EMT is mediated by SQSTM1. Images taken at 40x magnification. (Blue: DAPI, Green: Cytokeratin-18, Yellow, Vimentin).

Together, our findings suggest differential response to OS in AECs and CTCs of the fetal membranes, and this may be regulated in part via distinct autophagy activation (AECs) or inhibition (CTCs) and mediated by p38 MAPK leading to either senescence or EMT.

## Discussion

Understanding the changes in gene regulation that occur in fetal membranes during the onset of parturition is a growing field of research as membrane dysfunction and rupture leads premature ending of pregnancies. The distinct gene expression patterns underscore the potential involvement of autophagy in the pathogenesis of pPROM, emphasizing its relevance in understanding the underlying mechanisms of this condition. We also observed several lysosome-related genes downregulated in pPROM. An efficient autophagic response requires concomitant increase in lysosomal activity to ensure adequate degradation of substrates. Inadequate lysosomal biogenesis and degradation will result in decrease autophagic flux and autophagosome accumulation, which we postulate as a mechanism involved in membrane rupture, and untimely decrease in lysosomal activity may be a relevant pathology in premature rupture. Additionally in this study we have documented: 1) pathways other than senescence and EMT that can contribute to pPROM pathology, 2) mechanistic mediators of these pathways (e.g., p38MAPK), 3) cellular component that is vulnerable to these molecular activations. Any understanding of the mechanistic pathology related to pPROM will provide an opportunity for novel therapeutics in the future.

A discrepancy in the expression levels of LC3-I and LC3-II was noted when comparing TNIL and TL compared to prior studies. Specifically, we found no differences in LC3-I expression, but a decrease in LC3-II expression. In contrast, the previous studies reported an increase in LC3-I expression but no change in LC3-II expression [26]. It is important to note that changes in LC3B levels alone may not provide a direct indication of the overall autophagic activity. To address this limitation, we conducted a comprehensive examination of autophagy in both AECs and CTCs under OS conditions, the two distinct epithelial cell types in the fetal membranes. Interestingly, our findings revealed distinct responses between these two cell types. AECs exhibited a reduction in autophagic flux resulting in the accumulation of autophagosomes and accumulation of damaged or dysfunctional cellular components. Several diseases are associated with decreased autophagic flux such as neurodegenerative diseases [37,38], muscular dystrophy [39], and inflammatory diseases [40,41]. Damaged intracellular components accumulate and affect the function of cells and ultimately lead to cellular dysfunction. In contrast, CTCs exhibited an increase in autophagic flux, leading to enhanced consumption of autophagosomes. CTCs are able to withstand stress and have sufficient autophagic response even with prolonged exposure to OS. This supports the theory that the chorion acts as a “great wall of defense” against possible stressors from the maternal side by having higher tolerance to stress than the amnion, which is more vulnerable to OS-induced damage compared to CTCs. These differential responses highlight the cell-type-specific regulation of autophagy and provide valuable insights into the complex dynamics of autophagic processes under OS stress.

OS induced p38MAPK activation results in cellular senescence and EMT in AECs; however, the contribution of p38 MAPK in autophagy is not investigated. Phosphorylation of ULK1 at serine 555 by p38 MAPK has been reported and contributes to autophagy induction [42]. Our findings suggest an interplay between p38 MAPK and ULK1 in autophagosome formation. The specific activation of ULK1 at serine 555 is unique to AECs and absent in CTCs. ULK1 harbors multiple phosphorylation sites that can modulate its function, either activating or inhibiting it. The absence of this particular phosphorylation site offers valuable insights into the differential autophagic response observed between the two cell types. The loss of P-ULK1 in p38 MAPK KO cells suggests that p38 MAPK exerts its effects through this specific phosphorylation site in AECs.

Cell fate decisions are influenced by a variety of factors such as the source, type, and duration of stress signals. However, little is known about the factors that govern this decision-making process. Notably, both senescence and apoptosis have been linked to the stress-activated kinase p38 MAPK. This pathway is widely utilized by eukaryotic cells to interpret extracellular stimuli, particularly stress signals [43]. Upon activation, p38 MAPK phosphorylates various substrates that subsequently regulate diverse cellular functions [44]. p38 MAPK activation has been linked to the induction of both apoptosis and premature senescence [43,45–47] and we report these cell fates in AECs. [6,48]. Recent studies have shown that senescent cells may have impaired autophagy activity, which may contribute to the accumulation of dysfunctional organelles and proteins, leading to further cellular damage and tissue degeneration [49–51]. Autophagy is conventionally regarded as an anti-senescent process since it aids in maintaining cellular homeostasis by removing damaged macromolecules [52–56]. However, our findings indicate that autophagy induction can also lead to cellular senescence in AECs, suggesting that it is a pro-senescent process in fetal membrane cells. This paradoxical role of autophagy illustrates a complex interplay between the autophagy and senescence pathways. Studies have investigated a novel aspect of autophagy that appears to contradict its established role, revealing the presence of a pro-senescence function mediated by autophagy. During senescence, autophagy is activated and contributes to the synthesis of SASP by facilitating the formation of specialized cellular compartment called “target of rapamycin-autophagy spatial coupling compartment” (TASCC), where a high flux of amino acids and other metabolites is generated as a consequence of degradation of organelles and proteins through autophagy [49,57]. The formation of TASCC compartments through autophagy ensures the provision of essential components required for the production of SASP. This process allows senescent cells to effectively communicate with their microenvironment by secreting SASP, which can influence neighboring cells and contribute to various senescence-associated processes. Additionally, the elimination of autophagy-promoting factors, such as autophagy-related 7 and 12 and transcription factors [58], as well as inhibition of autophagic degradation of transcription factor GATA4 [59] has led to the development of senescence-like state, characterized by increased ROS [60].

While OS is known to induce irreversible EMT in AECs, EMT in CTCs has not yet been well-studied. EMT is a complex cellular process in which cells lose their epithelial characteristics and acquire mesenchymal properties, allowing them to migrate into surrounding tissues. We have reported EMT of AECs that can also cause inflammation and membrane dysfunction. Cellular senescence is linked to irreversible EMT in AECs via p38 MAPK mediated signaling [36] and amnion mesenchymal cells show pronounced inflammatory response contributing to the pathology[34][36]. The presence of chorion mesenchymal cells has been documented, but the pathways involved in EMT in CTCs remains unexplored. We demonstrated for the first time the induction of EMT in CTCs through the inhibition of autophagy, which is mediated by SQSTM1. Increased EMT may lead to disruption of the barrier function of the chorion trophoblast layer. This would allow decidual immune cells to infiltrate into the membrane and decrease its ability to stop microbial invasion. However, a limitation to this study is that TGF-β inhibitors are currently required to support CTC proliferation included in the media that could confound our data. Autophagy inhibition leading to EMT in CTCs seems to be TGF-β independent, though further studies are needed to tease out the interplay between EMT and autophagy. Additionally, there is limited research exploring the direct link between EMT and SQSTM1, but some studies have suggested that SQSTM1 may play a role in the EMT process in cancer cells. For example, a study by Bertrand et al. (2015) found that SQSTM1 downregulates expression of E-cadherin, occludin and claudin by increasing expression of SNAIL, which is a regulator of these junctional proteins [61]. Qiang et al. (2014) also showed that SQSTM1 promotes EMT and cell migration by stabilizing TWIST, a transcription factor involved in EMT [62]. However, more research is needed to determine the specific role of SQSTM1 in EMT in different contexts, including in fetal membranes.

**Figure 9.**
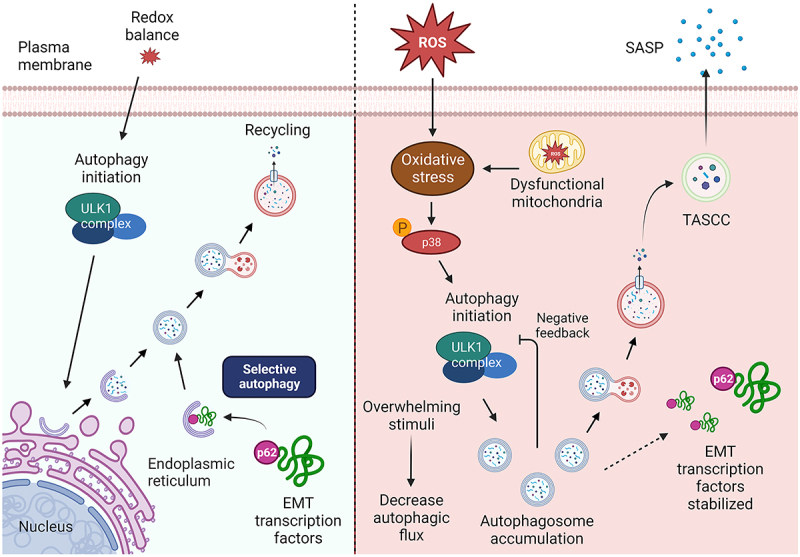
Mechanistic pathways of cell fate determination by autophagy

Here we have shown that autophagy determines cell fate differently in two different cell types of the same tissue, which further emphasizes the complexity of the fetal membranes and its function. We demonstrate that autophagy induction by p38 MAPK signaling drives AECs to preferentially enter senescence. On the chorion side, autophagy induction has limited effects on CTCs, whereas autophagy inhibition can induce EMT and inflammation. The proper balance of autophagy is necessary for maintaining the homeostasis of fetal membrane cells. Tipping the balance to either excessive inhibition or induction can disrupt fetal membrane homeostasis. This can occur either by inducing EMT on the chorion side or by promoting senescence and SASP factors on the amnion side, resulting in an inflammatory phenotype. A visual representation of these findings are shown in Figure 9.

(Left) Under cellular homeostasis, the autophagy machinery plays a critical role in maintaining redox balance by damaged intracellular components. Moreover, SQSTM1/p62-mediated selective autophagy ensures the proper turnover of various regulators and transcription factors, including those involved in epithelial-mesenchymal transition (EMT), thus preserving the epithelial phenotype. (Right) In conditions of increased oxidative stress, the sustained activation of p38 MAPK triggers the initiation of autophagy. However, when the stimuli become overwhelming, it can lead to the accumulation of autophagosomes and a decrease in autophagy flux. This accumulation creates a negative feedback loop that further exacerbates the buildup of damaged components within the cell. As a consequence, EMT transcription factors may become stabilized and retain their functionality, as they are no longer targeted for degradation by selective autophagy. Furthermore, autophagy may also play a role in promoting cellular senescence by generating target of rapamycin-autophagy spatial coupling compartment (TASCC) to facilitate the production of senescence-associated secretory phenotype (SASP) factors.

## Materials and methods

### Institutional review board approval

The study was approved by the Institutional Review Board of their respective institutions: University of Texas Medical Branch (UTMB) at Galveston, TX (UTMB 11-251) and University Hospital Hradec Kralove, Czech Republic (200804 SO1P), in compliance with all relevant Federal regulations governing the protection of human subjects.

### Collection of human fetal membrane samples

Fetal membrane (amniochorion) samples from four groups were collected from John Sealy Hospital, UTMB at Galveston, Texas, and University Hospital Hradec Kralove, Czech Republic: 1) TNIL – term pregnancies (37 - 40 weeks of gestation) with no labor, who underwent elective Caesarian section; 2) TL - term pregnancies, with spontaneous labor, subsequent spontaneous membrane rupture and normal vaginal delivery; and 3) pPROM - preterm pregnancies with absence of labor prior to spontaneous rupture of membranes. Membranes from women with pregnancy complications such as multiple gestations, placenta previa, preeclampsia, eclampsia, and fetal anomalies were excluded from the study. Amniochorion were dissected from the midzone portion of the membranes and washed with normal saline. Blood clots and decidua were thoroughly removed using a cotton gauze. Six-millimeter punch biopsies were placed in 10% neutral buffered formalin for immunohistochemistry. Biopsies were placed in RNAlater® solution for sequencing studies, or snap-frozen for western blot analyses.

### Gene expression analysis and Ingenuity Pathway Analysis

Differentially expressed genes (DEG) were identified using the CLC Genomics Workbench (version 22.0.2, QIAGEN Bioinformatics). Differentially expressed genes (DEGs) were defined as those with an adjusted *p*-value ≤ 0.05 and a fold change ≥ 1.5. Ingenuity Pathway Analysis (IPA, QIAGEN Bioinformatics) software was employed to investigate the biological functions and pathways associated with these genes. The list of DEGs (adjusted *p*-value ≤ 0.05 and a fold change ≥ 1.5), along with their respective fold change and adjusted *p*-values, were imported into IPA using CLC Genomics. All DEGs were annotated based on their ENSEMBL ids. We identified autophagy genes using a comprehensive autophagy transcriptional toolbox developed by Bordi and colleagues to get useful insights about the autophagy status in the different pregnancy conditions [18]. A core expression analysis was performed on the imported DEGs. The list of DEGs (FDR adjusted *p*-value ≤ 0.05 and a fold change ≥ 1.5), along with their respective fold change and adjusted *p*-values, were imported into IPA® using CLC Genomics. The expression log ratio was used to calculate the directionality (z-scores) of identified features. The core analysis provides functional annotations, canonical pathways, and molecular networks associated with the DEGs based on the Ingenuity Knowledge Base (direct and indirect relationships), a comprehensive repository of biological interactions and functional annotations.

### Culture of immortalized human fetal membrane cells

Immortalized human amnion epithelial cells (hFM-AEC) and chorion trophoblast cells (hFM-CTC) cell lines were used in this study. These cell lines were previously characterized and validated to model fetal membrane cells [32]. hFM-AECs were cultured in complete human keratinocyte serum-free media (KSFM), with bovine pituitary extract (30 μg/mL), epidermal growth factor (0.1 ng/mL), and primocin (0.5 mg/mL). hFM-CTCs were cultured in DMEM/F12 supplemented with 0.20% FBS, 0.1 mM β-mercaptoethanol, 0.5% penicillin/streptomycin, 0.3% BSA, 1× ITS-X, 2 μM CHIR99021, 0.5 μM A83-01, 1 μM SB431542, 1.5 μg/mL L-ascorbic acid, 50 ng/mL epithelial growth factor, 0.8 mM VPA, and 1× Y27632 (Rock inhibitor). All cells were grown at 37 °C and 5% CO_2_ environment until 80-90% confluency was achieved.

### Treatment preparation

CSE was prepared by bubbling smoke drawn from a single lit commercial cigarette through 25 mL of cell culture medium (Ham’s F12/DMEM mixture with antimicrobial agents) as previously described [63]. The extract was filter sterilized through a 0.22 mm Millipore filter to remove contaminant microbes and insoluble particles. A 1:50 and 1:100 dilutions of CSE to cell culture media were prepared fresh prior to treatment. Bafilomycin A1 and torin-1 were reconstituted in dimethylsulfoxide and diluted in cell culture medium resulting to 100 nM and 30 nM concentrations, respectively. Cells were treated with CSE, Baf, or Tor in culture media and incubated at 37°C and 5% CO_2_ environment for 3-48 hours.

### Western blotting

Biopsies of fetal membranes or cell cultures were lysed in radioimmunoprecipitation lysis buffer [50 mM Tris (pH 8.0), 150 mM NaCl, 1% Triton X-100, and 0.5% sodium deoxycholate, and 0.1% SDS] supplemented with a protease and phosphatase inhibitor cocktail and phenylmethylsulfonyl fluoride. Tissues were homogenized using 0.5mm ZrO beads in Next Advance Bullet Blender. Cell culture lysates were collected after scraping the culture plate. The lysis mixtures were vortexed for 10 sec, sonicated for 30 sec, and kept on ice for 10 min. Insoluble material were removed by centrifugation at 10,000 rpm for 20 min at 4°C. The concentration of protein in each lysate were determined using the BCA protein assay kit (Pierce BCA Protein Assay Kit, Thermo Scientific). Equal protein (15 μg for cell lysates, 30 μg for tissues) from each sample were loaded onto a 12% SDS-PAGE gel and electrophoresed at 120 V. The resolved proteins were transferred to a PVDF membrane using the iBlot transfer apparatus (Bio-Rad Laboratories). The membranes were blocked using Tris-Buffered Saline (TBS) containing 0.1% Tween 20 (TBS-T) and 5% skim milk or bovine serum albumin for 1h at room temperature. Membranes were probed with LC3B (1:1000, PA1-46286, Invitrogen), SQSTM1 (1:500, PA5-27247, Invitrogen), ULK1 (1:500, 8054, Cell Signaling), P-ULK1 S555 (1:500, 5869T, Cell Signaling), ATG13 (1:500, PA5-75682, Invitrogen), p38 MAPK (1:1000, 9212S, Cell Signaling), P-p38 MAPK Thr180/Tyr182 (1:400, 9211S, Cell Signaling), N-cadherin (1:300, ab98952, Abcam), E-cadherin (1:300, ab15148, Abcam), or β-actin (1:15000, ab49900, Abcam) antibodies overnight at 4°C. The membranes were incubated with appropriate anti-rabbit (1:10000, NA934VS, Amersham) or anti-mouse (1:10000, NA931VS, Amersham) peroxidase-conjugated IgG secondary antibody for 1 hr at room temperature. All blots were developed using ECL chemiluminescence reagents and a Bio-Rad Chemidoc imaging system. Detected bands were analyzed densitometrically using the Image J software and results were normalized to β-actin expression.

### Quantitative real-time PCR

To determine the expression levels of CDKN1A after treatment, cells were collected and lysed using lysis buffer (Qiagen). RNA was extracted using a RNeasy kit (Qiagen) per the manufacturer’s instructions. Total RNA (2 μg) was reverse-transcribed using a High-Capacity RNA-to-cDNA Kit (Applied Biosystems). qRT-PCR reactions were performed using TaqMan™ Universal PCR Master Mix (Applied Biosystems) and TaqMan™ Gene Expression Assay (FAM) probes for CDKN1A (Hs00355782_m1) with GAPDH (Hs00177504_m1) as internal control. The results of qRT-PCR assays were presented as mean of three independent RNA preparations. Changes in gene expression levels were calculated using the ΔΔCt method.

### Immunocytochemistry

Immunocytochemical staining for LC3 (1:200, CAC-CTB-LC3-2-IC, Cosmo Bio), SQSTM1 (1:300, PA5-27247, Invitrogen), vimentin (1:300, ab195878, Abcam) and cytokeratin-18 (1:300, ab194124, Abcam) were performed for different experimental endpoints. After treatment, cells were fixed with 4% paraformaldehyde, permeabilized with 0.5% Triton X, and blocked with 3% BSA in PBS prior to overnight incubation with primary antibodies at 4°C. After washing with PBS, slides were incubated with appropriate secondary antibodies diluted in PBS (Alexa Fluor 488 conjugated anti-rabbit IgG [1:1000, ab150073, Abcam] or Alexa Fluor 594 conjugated anti-mouse IgG [1:1000, A11005, Invitrogen]). Slides were incubated for 1 hour in the dark. Slides were washed with PBS then treated with NucBlue™ Fixed Cell ReadyProbes™ Reagent (Invitrogen) and then mounted using Mowiol 4-88 mounting medium (Sigma Aldrich).

### p38 MAPK knockout by CRISPR/Cas9

p38 MAPK knockout (p38 KO) in hFM-AEC and hFM-CTC by using Clustered Regularly Interspaced Short Palindromic Repeats (CRISPR)/CRISPR associated protein 9 (Cas9) gene-editing technique were previously generated and validated [10,11]. Multi-guide sgRNAs for MAPK14 (p38α) and Cas9 2NLS nuclease were purchased (Synthego Corp.) and used for transfection according to the manufacturer’s instruction. Transfection was facilitated using Lipofectamine™ CRISPRMAX™ Transfection Reagent (CMAX00015, Invitrogen). The transfected pooled cells were analyzed for p38 KO efficiency with Western blot for p38 expression.

### Histochemical detection of senescence-associated β-galactosidase activity

Senescent cells were identified using a histochemical staining kit (CS0030, Sigma-Aldrich) per manufacturer’s instructions. After 48 hours of exposure to corresponding treatments, cells were washed twice with PBS, fixed for 6-7 min with the provided Fixation Buffer, washed again with PBS, and incubated for 4 hours at 37°C with fresh β-gal solution. Following incubation, blue cells were visualized using a standard light microscope. Four regions of interest were captured per treatment group using bright-field microscopy at 40x magnification.

### Flow cytometry analysis for senescence

To quantitatively measure the senescent cells, senescence-associated β-galactosidase activity was evaluated using flow cytometry. After 48 hours of incubation with treatment, cells were incubated for one hour in growth medium supplemented with 100 nM bafilomycin A1. Cells were incubated with 6 μM of 5-Dodecanoylaminofluorescein di-β-D-Galactopyranoside (C_12_FDG, eBioscience) for one hour at 37°C. Cells were collected, washed with PBS and resuspended in DNA prep stain containing propidium iodide (PI) to select for viable cells. Samples were run immediately on the CytoFlex flow cytometer (Beckman Coulter). Cells treated with DMSO only (vehicle) were used as negative controls for gating while C_12_FDG only and PI only treated cells were used to compensate for FITC and PE signals. After gating for viable, single cells, data analysis was performed using FlowJo™ (BD Biosciences). Percent positive cells for C_12_FDG fluorescence were calculated and compared to control.

### Cell Shape Index Analysis

Cell shape index was determined by evaluating one frame for each replicate (total of 3 replicates) per treatment for cell circularity using ImageJ software. All cells that are within the frame were outlined using the polygon tool. The shape index was calculated using the formula SI = 4π*Area/Perimeter^2^, which is an established method that was originally reported to determine vascular cell shape [64].

### siRNA transfections

SQSTM1 was downregulated using ON-TARGETplus™ siRNA (Dharmacon, Horizon Discovery Ltd.). CTC cells were cultured to nearly 50% confluence. Prior to siRNA transfection, cells were incubated with anti-microbial-free media overnight. Cells were then incubated for 6 hours with siRNA complexes, which were freshly prepared 100 nM siRNA to SQSTM1 or Non-targeting (NT) siRNA as control, and 0.3% Lipofectamine RNAiMAX (Invitrogen) in Opti-MEM I Reduced Serum Medium. Cells were further incubated in growth medium for 24 hours before treatment. Downregulation efficiency of SQSTM1 was validated by Western blot.

### Statistical analysis

Data were presented as means ± SEM. Comparison between two groups were analyzed using two-tailed Student’s t tests. One-way ANOVAs with general linear model procedures using a univariate approach were applied for more than two groups. P values were adjusted for multiple hypothesis testing using false discovery rate. All statistical analyses will be performed with GraphPad Prism. A *p*-value of > 0.05 was considered significant.

## Abbreviations

AEC: Amnion epithelial cell
AMC: Amnion mesenchymal cell
CSE: Cigarette smoke extract
CTC: Chorion trophoblast cell
DEG: Differentially expressed genes
EMT: Epithelial-to-mesenchymal transition
OS: Oxidative stress
p38 MAPK: p38 mitogen-activated protein kinase
pPROM: Preterm premature rupture of membranes
ROS: Reactive oxygen species
SASP: Senescence-associated secretory phenotype
TL: Term in labor
TNIL: Term not in labor

## Supplementary Material

Supplementary Figures and Tables.docx

## Declaration statement

IUM serves on the scientific advisory board of Luca Biologics.
