# Supplementary material for: Autophagy Determines Distinct Cell Fates in Human Amnion and Chorion Cells
Source: Autophagy Rep. 2024 Feb 7;3(1):2306086. doi: 10.1080/27694127.2024.2306086 (PMC10871702; doi:10.1080/27694127.2024.2306086)
Supplement: Supplementary Figures and Tables.docx [file KAUO_A_2306086_SM8904.docx]

# Supplementary Figures and Tables


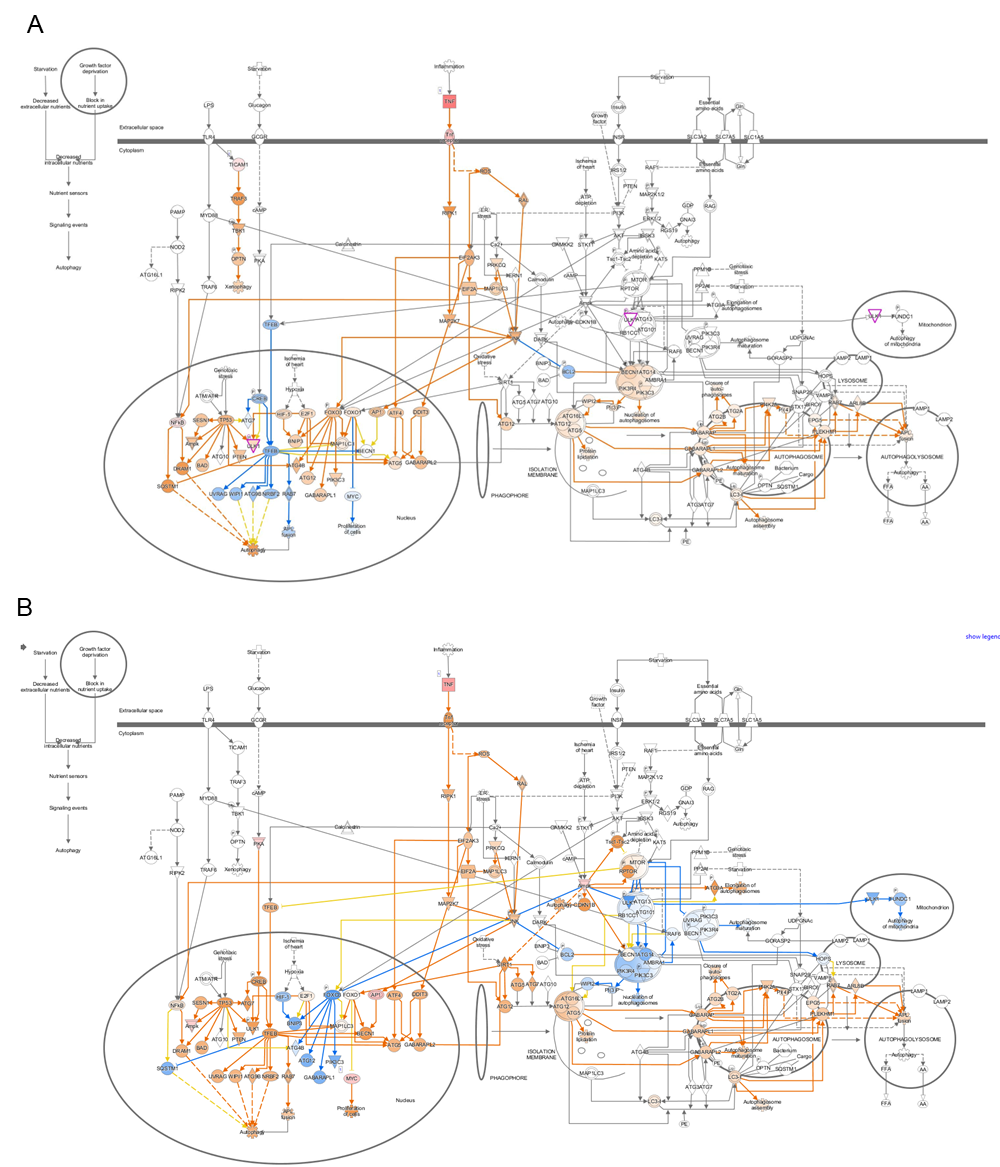


Supplementary Figure 1. Autophagy canonical pathway by Ingenuity Pathway Analysis in (A) term in labor and (B) preterm premature rupture of membranes. Legend: orange = predicted activation, blue = predicted inhibition, yellow = findings inconsistent with state of downstream molecule, gray = effect not predicted, red = increased gene expression.


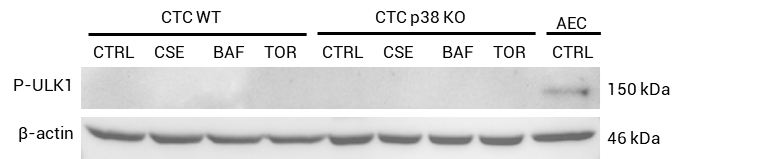


**Supplementary Figure 2**. Western blot analysis showed that P-ULK1 at Ser555 was not present in CTCs. AEC was shown as a positive control.


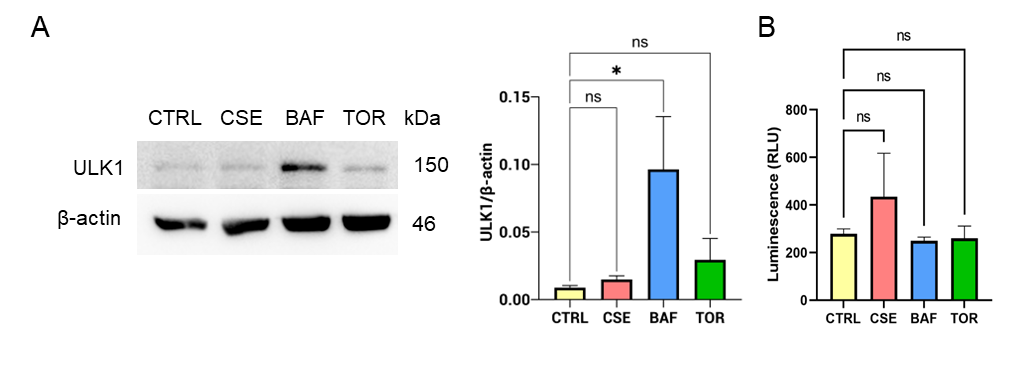


**Supplementary Figure 3.** Western blot analysis showed that autophagy inhibition increases ULK1 levels but not oxidative stress (CSE) or autophagy induction (TOR) in chorion trophoblast cells. Error bars represent mean ± SEM. (*: p <0.05, ns: not significant, CSE: cigarette smoke extract, BAF: Bafilomycin A1, TOR: Torin-1)


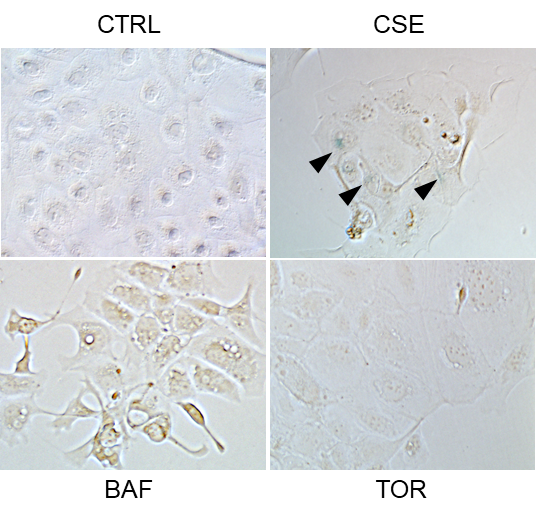


**Supplementary Figure 4.** Histochemical detection of senescence-associated beta-galactosidase (SA-β-Gal) activity exhibited blue staining in senescent cells (arrowheads) in chorion trophoblast cells upon oxidative stress (CSE) but not autophagy inhibition (BAF) or induction (TOR). (CSE: cigarette smoke extract, BAF: Bafilomycin A1, TOR: Torin-1)


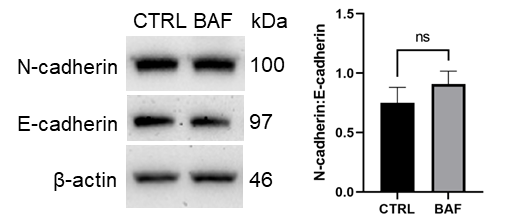


**Supplementary Figure 5**. Western blot analysis did not show changes in N-cadherin:E-cadherin ratio upon autophagy inhibition (BAF) in amnion epithelial cells (ns: not significant, BAF: Bafilomycin A1).
